# Correction: Learning precise spatiotemporal sequences via biophysically realistic learning rules in a modular, spiking network

**DOI:** 10.7554/eLife.87507

**Published:** 2023-03-13

**Authors:** Ian Cone, Harel Z Shouval

**Keywords:** None

Cone I, Shouval HZ. 2021. Learning precise spatiotemporal sequences viabiophysically realistic learning rules in a modular, spiking network. *eLife* **10**:e63751. doi: 10.7554/eLife.63751.

Published 18 March 2021

A recent publication (Zajzon et al., 2023), which reproduced this paper’s Markovian sequence model in NEST (Zajzon et al., 2022), has made us aware of an error in the original code included in the data availability. Specifically, the original Markovian code (which we hereafter refer to as Markovian FF) improperly restricted Messenger to Timer intercolumnar connections such that they were limited to be feed-forward (i.e. ordinal to the presented sequence). We thank Zajzon et al., 2023 for also proposing a fix (via raising the feed-forward threshold, as described below). An updated version of the Markovian code (which we hereafter refer to as Markovian A2A), which correctly allows for any (all-to-all or A2A) Messenger to Timer intercolumnar connections, is now included (along with the previous code, MATLAB FF) in the ModelDB upload. Supplementary File 1 and 2 have been updated to include accurate and correctly scaled values, for both Markovian FF and Markovian A2A. Additionally, an inconsistency in Equations 8 and 9 has been corrected, and text in the Materials and methods has been added to highlight the importance of learning thresholds r_th_ and r_th_^FF^. We thank the authors of Zajzon et al., 2023 for their contributions to recognizing and rectifying these errors.

The corrected parameters in Markovian A2A are as follows:

· Feed-forward learning threshold r_th_^FF^ = 30Hz

· Saturation level for feed-forward LTD eligibility trace T_d_^max,FF^ = .0045

· Activation rate for feed-forward LTP eligibility trace h_p_^FF^ = 8.8 x 3500 ms^-1^

· Activation rate for feed-forward LTD eligibility trace h_d_^FF^ = 10 x 3500 ms^-1^

· Feed-forward learning rate h^FF^ = 0.4

The original parameters in Markovian FF were set as:

· Feed-forward learning threshold r_th_^FF^ = 20Hz

· Saturation level for feed-forward LTD eligibility trace T_d_^max,FF^ = .00345

· Activation rate for feed-forward LTP eligibility trace h_p_^FF^ = 20 x 3500 ms^-1^

· Activation rate for feed-forward LTD eligibility trace h_d_^FF^ = 15 x 3500 ms^-1^

· Feed-forward learning rate h^FF^ = 0.25

The corrected equations 8 and 9 read the following:

$$\tau^{p}\frac{dT_{ij}^{p}}{dt}=-T_{ij}^{p}+{\eta^{\text{p}}H}_{ij}\left(T_{max}^{p}-T_{ij}^{p}\right)(8)$$

$$\tau^{d}\frac{dT_{ij}^{d}}{dt}=-T_{ij}^{d}+{\eta^{\text{d}}H}_{ij}\left(T_{max}^{d}-T_{ij}^{d}\right)(9)$$

The original equations 8 and 9 were published as:

$$\tau^{p}\frac{dT_{ij}^{p}}{dt}=-T_{ij}^{p}+{\eta^{\text{p}}H}_{ij}\frac{\left(T_{max}^{p}-T_{ij}^{p}\right)}{T_{max}^{p}}(8)$$

$$\tau^{d}\frac{dT_{ij}^{d}}{dt}=-T_{ij}^{d}+{\eta^{\text{d}}H}_{ij}\frac{\left(T_{max}^{d}-T_{ij}^{d}\right)}{T_{max}^{d}}(9)$$

The following text has been appended to the Materials and methods:

“Our Hebbian term is also subject to rate thresholds r_th_ and r_th_^FF^ in the recurrent and feed-forward cases, which we further discuss at the end of this section.”

“However, in the presence of noise, modules can have spurious activity overlaps which cause non-zero H_ij_ and therefore potentiation of weights W_ij_ which are non-sequential. This can lead to network instability and a failure to encode the presented sequence. To account for this, rate thresholds r_th_ and r_th_^FF^ are included in the Hebbian term H_ij_. By setting these thresholds above the effective noise level (see Supplementary File 1), the Hebbian overlap H_ij_ used to activate traces ignores the random, noise-driven overlaps. Crucially, as r_th_^FF^ dictates the sensitivity to inter-columnar overlaps, it is a critical and necessary parameter to enable the all-to-all connectivity of the model.”

The article has been corrected accordingly.

## Additional files

Supplementary file 1. Table of main model parameters.

Supplementary file 2. Table of reservoir, sparse net, and rate-based model parameters.
