# Supplementary material for: Learning precise spatiotemporal sequences via biophysically realistic learning rules in a modular, spiking network
Source: eLife. 2021 Mar 18;10:e63751. doi: 10.7554/eLife.63751 (PMC7972481; doi:10.7554/eLife.63751)
Supplement: Supplementary file 1. — For full code, see http://modeldb.yale. [file elife-63751-supp1.docx]

| Parameter | Value | Units | Description |
| --- | --- | --- | --- |
| N | 100 | - | Number of neurons per population |
| dt | 1 | ms | Integration time step |
| T | 50 | ms | Stimulus pulse duration |
| τ_stim_ | 50 | ms | Decay constant of stimulus |
| τ_w_ | 40 | ms | Time window for firing rate integration |
| p_r_ | 30 | Hz | Rate of Poisson stimulus pulse |
| $\boldsymbol{\sigma}_{\mathbf{N}}$ | Ɲ (0,100) |  | Gaussian white noise at membrane |
| ρ | 1/7 | - | Fractional change of synaptic activation |
| τ_s_^E^, τ_s_^I^, τ_s_^inp^ | 80,10, 10 | ms | Time constant for synaptic activation for excitatory (EE and IE), inhibitory (EI), and input connections |
| g_L_ | 10 | nS | Leak conductance |
| C_m_ | 200 | pF | Membrane capacitance |
| E_L_ | -60 | mV | Leak reversal potential |
| E_E_, E_I_ | -5,-70 | mV | Excitatory and inhibitory reversal potentials |
| v_th_, v_th_^I^ | -55, -50 | mV | Spiking threshold potential (excitatory, inhibitory) |
| v_rest_ | -60 | mV | Resting potential |
| v_hold_ | -61 | mV | Reset potential |
| t_ref_ | 3 | ms | Absolute refractory period |
| τ_p_, τ_d_ | 2000, 1000 | ms | LTP/LTD eligibility trace time constant, recurrent connections |
| T_p_^max^, T_d_^max^ | 0.0033, 0.00345 | - | Saturation level, LTP/LTD eligibility trace, recurrent connections |
| η_p_, η_d_ | 45 x 3500, 25 x 3500 | ms^-1^ | Activation rate, LTP/LTD eligibility trace, recurrent connections |
| τ_p_^FF^, τ_d_^FF^ | 200, 800 | ms | LTP/LTD eligibility trace time constant, feed forward connections |
| T_p_^max,FF^ | 0.0034 | - | Saturation level, LTP eligibility trace, feed forward connections |
| T_d_^max,FF^ | 0.00345 (MARKOVIAN FF) 0.0045 (MARKOVIAN A2A) | - | Saturation level, LTD eligibility trace, feed forward connections |
| η_p_^FF^ | 20 x 3500 (MARKOVIAN FF)  8.8 x 3500 (MARKOVIAN A2A) | ms^-1^ | Activation rate, LTP eligibility trace, feed forward connections |
| η_d_^FF^ | 15 x 3500 (MARKOVIAN FF)  10 x 3500 (MARKOVIAN A2A) | ms^-1^ | Activation rate, LTD eligibility trace, feed forward connections |
| r_th_ | 10 | Hz | Hebbian activation threshold (recurrent connections) |
| r_th_^FF^ | 20 (MARKOVIAN FF)  30 (MARKOVIAN A2A) | Hz | Hebbian activation threshold (feed forward connections) |
| T_reward_ | 25 | ms | Duration of neuromodulator presentation upon change in stimulus |
| T_tr_ | 25 | ms | Duration of refractory period for traces following neuromodulator presentation |
| d_reward_ | 25 | ms | Novelty delay upon change in stimulus |
| η | 0.16(recurrent)  20 (feed-forward, MARKOVIAN FF)  32 (feed-forward, MARKOVIAN A2A) | ms^-1^ | Learning rates, recurrent and feed forward connections (note that these are scaled by the delay period, so are implemented in MATLAB as η = 2*η_fixed_ / T_reward_). Slower learning rates will be more stable but take more trials to converge to fixed-points. |
| ϕ | 0.26 (0.3) | - | Sparsity of fixed connections, implemented in MATLAB as 0.3, which results in an effective sparsity of 0.26 because of random number generator oddities |
| W_EE_^MT^, W_EI_^MT^ | 0.2, -70 | nS | Synaptic connection strength, Timer to Messenger excitatory to excitatory (EE) and inhibitory to excitatory (EI) connections |
| W_EI_^TT^, W_EI_^MM^ | -100, -100 | nS | Synaptic connection strength, intercolumnar Timer-Timer and Messenger-Messenger inhibitory to excitatory (EI) connections |
| W_IE_^TT^, W_IE_^MM^ | 0.2, 1 | nS | Synaptic connection strength, intracolumnar Timer-Timer and Messenger-Messenger excitatory to inhibitory (IE) connections |

**Supplementary File 1. Table of Main Model Parameters.** For full code, see http://modeldb.yale.edu/266774
