# Supplementary material for: Learning precise spatiotemporal sequences via biophysically realistic learning rules in a modular, spiking network
Source: eLife. 2021 Mar 18;10:e63751. doi: 10.7554/eLife.63751 (PMC7972481; doi:10.7554/eLife.63751)
Supplement: Supplementary file 2. [file elife-63751-supp2.docx]

| Parameter | Value | Units | Description |
| --- | --- | --- | --- |
| K | 280 | - | Number of units in reservoir |
| g | 1.5 | - | Gain parameter |
| M | 1120 | - | Number of units in sparse pattern net |
| $\theta_{m}$ | 7.5 | Hz | Threshold for columnar to reservoir excitation |
| $\theta_{o}$ | 0.1 | - | Threshold for reservoir to sparse net excitation |
| $Q_{\max}$ | 15 | nS | Max feedback weight |
| $\tau_{\mathrm{net}}$ | 100 | ms | Time constant for units in reservoir |
| $\tau_{u}, \tau_{\mathrm{ui}}$ | 40,10 | ms | Excitatory and inhibitory time constants, rate-based network |
| $u_{c}$ | 2 | - | Upper threshold, rate-based transfer function |
| $\theta$ | 0 | - | Lower threshold, rate-based transfer function |
| $\nu$ | 2 | - | Scaling parameter, rate-based transfer function |

**Supplementary File 2. Table of Reservoir, Sparse Net, and Rate-Based Model Parameters.** For full code and full list of non-Markovian parameters, please refer to http://modeldb.yale.edu/266774
